# Association Between CT With Angiography and 30-Day Risk of Stroke or Transient Ischemic Attack in a Canadian Emergency Department Setting

**DOI:** 10.7759/cureus.74041

**Published:** 2024-11-19

**Authors:** Johanna L Jacob, Urouj Rashid, Chel H Lee, Bijoy K Menon, Katie Lin

**Affiliations:** 1 Neuroscience, University of Alberta, Edmonton, CAN; 2 Biological Sciences, University of Calgary, Calgary, CAN; 3 Critical Care Medicine, University of Calgary, Calgary, CAN; 4 Clinical Neurosciences, University of Calgary, Calgary, CAN; 5 Radiology, University of Calgary, Calgary, CAN; 6 Community Health Sciences, University of Calgary, Calgary, CAN; 7 Emergency Medicine, University of Calgary, Calgary, CAN

**Keywords:** emergency medicine, neuroimaging, stroke, transient ischemic attack, vertigo

## Abstract

Introduction: A subset of undifferentiated vertigo cases can be attributed to dangerous central causes such as posterior circulation ischemic stroke (PCIS) or transient ischemic attack (TIA). Due to a lack of validated clinical risk scoring tools, there is currently high heterogeneity in emergency department (ED) neuroimaging practices for patients presenting with undifferentiated vertigo. Therefore, this study assessed the utility of head and neck CT with angiography (CTA) for risk stratifying ED patients presenting with vertigo. The primary objective of this study was to compare 30-day stroke and TIA outcomes between ED vertigo patients who received CTA at their index visit versus those who did not. The impact of index visit CTA on secondary outcomes of interest was also measured, including ED length of stay (LOS), hospital LOS, and 30-day ED revisit rate.

Methods: This retrospective study analyzed ED visit data across four tertiary care ED's over a one-year period. Adult patients presenting with a chief complaint of vertigo were eligible for study inclusion. Administrative data of the variables of interest was gathered from Canadian medical databases. Regression modeling was used to adjust for predetermined variables to evaluate the association between index visit CTA imaging, and stroke or TIA diagnosis at 30 days.

Results: A 30-day diagnosis for stroke or TIA was found in 20.7% of the CTA group, and in 1.2% of the No CTA group. The odds ratio (OR) was 22.3 (95% confidence interval (CI): 15.03-33.02) unadjusted, and 18.3 (95% CI: 14.85-22.45) after adjustment. The CTA group had a longer average ED LOS (+114 minutes), a shorter average total hospital LOS within 30 days (-2.2 days), and a higher 30-day ED revisit rate when compared to the No CTA group (4.0% versus 1.5%).

Conclusions: Patients who received CTA at their index visit had 18.3 times greater odds of TIA or stroke diagnosis at 30-days, stayed longer in the ED, were more likely to revisit the ED within 30 days, and had a shorter mean hospital stay.

## Introduction

Vertigo is a common yet challenging emergency department (ED)-presenting complaint, accounting for approximately 4% of all ED visits [1]. The majority of vertigo presentations are due to benign peripheral causes, such as benign paroxysmal positional vertigo and vestibular neuritis. However, an estimated 3.2-6.5% of undifferentiated dizziness and vertigo cases are the result of central nervous system causes such as posterior circulation ischemic stroke (PCIS) or transient ischemic attack (TIA) [2,3]. PCIS or TIA leading to vertigo occurs due to ischemia affecting posterior structures of the brain supplied by the vertebrobasilar system, including the cerebellum and brainstem, which regulate equilibrium and balance [4].

Early identification and management of PCIS and TIA are associated with better functional outcomes for patients, yet risk stratification of this population in the ED remains difficult. Prior work, such as the Head Impulse Nystagmus and Test of Skew (HINTS) exam, has focused largely on clinical assessment for risk stratification [5]. However, literature suggests that the accuracy of the HINTS exam may be lower when performed by emergency physicians as compared to neuro-ophthalmology specialists [5]. Furthermore, current TIA scoring methods, such as the ABCD2/3 scores and the Canadian TIA score, focus more on anterior circulation symptoms and may not be as helpful for PCIS [6,7]. Diagnostic imaging remains a heavily relied upon adjunct for vertigo risk stratification in the ED. Although MRI is the gold standard imaging modality, cost and resource barriers limit its accessibility in the ED environment. While non-contrast CT (NCCT) and CT with angiography (CTA) are more accessible alternatives to MRI, they have lower diagnostic accuracy for detecting PCIS [8]. It has been suggested that vascular abnormalities detected on CTA may be useful for identifying patients at higher short-term risk of PCIS and TIA [9]. However, little is currently known about the impact of CTA use on patient-centered outcomes.

The purpose of this study was to understand how CTA is currently being utilized in undifferentiated ED vertigo patients and whether there was a predictive association of CTA with short-term patient-oriented outcomes.

Preliminary findings of this work were previously presented as a meeting abstract at the University of Calgary’s 2023 Department of Emergency Medicine Research Day on May 4, 2023.

## Materials and methods

Study design and population

This study analyzed ED visit data across four tertiary care ED’s in Calgary, Alberta, including: Foothills Medical Centre (FMC), Peter Lougheed Center (PLC), Rockyview General Hospital (RGH), and South Health Campus (SHC). Adult patients (18 years or older) who presented to one of these ED’s between January 1, 2021 and January 1, 2022 with a Canadian Emergency Department Information System (CEDIS) chief complaint of vertigo (CEDIS code 403) were eligible for study inclusion. Patients were excluded if they were unidentified in the system, had no valid universal lifetime identifier (ULI) number, were non-Alberta residents, were transferred directly from another acute treatment healthcare facility (i.e. urgent care, hospital, or ED), or left against medical advice (AMA).

This retrospective observational cohort study utilized administrative data from linked Canadian electronic medical record databases (including ED, inpatient, and outpatient clinic databases). Physician orders for neuroimaging in this cohort was based on individual clinician judgement.

The primary objective of this study was to compare 30-day stroke and TIA outcomes between two groups: ED vertigo patients who received CTA at their index visit (CTA group), and those who did not (No CTA group) (Appendix 1). Secondary objectives were to evaluate the impact of index visit CTA on ED length of stay (LOS), number of repeat ED revisits, and total hospital LOS (including admission), all within 30 days of the index ED visit.

Data analysis

Following an electronic health record capture of patients presenting to the ED with a chief complaint of vertigo during the specified period, duplicate and incomplete records were removed. A generalized linear regression model was used to calculate odds ratios (ORs) for the primary outcome of interest (30-day stroke and TIA) and to adjust for predefined covariates of interest within the study population. The covariates of interest were defined a priori as follows: age (categorical: <50, 50-75, >75), sex (binary), previous stroke (binary), Canadian Triage and Acuity Scale (CTAS) score (binary: 1-2 vs 3-5), ambulance arrival (binary), site of index ED visit (binary: comprehensive stroke center vs non-comprehensive stroke center), systolic blood pressure (categorical in mmHg: <90, 90-140, 140-185, >185), and additional imaging orders at index visit (NCCT, MRI, magnetic resonance angiography (MRA)) (all binary). Stepwise forward selection and multicollinearity testing were used to identify factors most predictive of stroke during modelling analysis. Variables considered for model input were determined a priori based on clinical rationale and existing stroke and TIA risk stratification score elements that were reliably available for analysis within the electronic health database. ORs for 30-day stroke or TIA diagnosis were calculated with 95% CIs. All statistical analysis was completed using R statistical software (version 4.0.1).

## Results

Study population baseline characteristics

Total 3,455 patients were included in the study with 699 in the index-visit CTA group and 2,756 in the No CTA group. Baseline characteristics are reflected and summarized in Table 1. Overall, the distribution of CTAS scores and triage vital signs were similar in both groups. The CTA group had higher mean age, higher incidence of previous stroke, higher proportion presenting to a comprehensive stroke center ED rather than a community ED, more arrival by ambulance, higher incidence of concurrent MRI’s ordered, higher proportion of hospital admission at index visit, and higher rate of both stroke consults and Stroke Prevention Clinic referrals. 19.4% of the No CTA group had some form of alternative neuroimaging completed at index visit (most commonly NCCT).

**Table 1:** Baseline characteristics and summary of the study population CTA: CT with Angiography; CTAS: Canadian Triage and Acuity Scale; ED: Emergency department; TIA: Transient ischemic attack; FMC: Foothills Medical Centre; NCCT: Non-contrast CT; MRA:

Characteristic	CTA at Index Visit, N= 699	No CTA at Index Visit, N= 2,756
Age, Mean ± SD	64 ± 16	55 ± 19
Female Sex, N (%)	397 (54.2)	1,703 (61.8)
Clinical History		
Previous Stroke or TIA, N (%)	71 (10.2)	144 (5.2)
Index Hospital Type		
Comprehensive Stroke Centre (FMC), N (%)	258 (36.9)	702 (25.5)
Non-Comprehensive Stroke Centre, N (%)	441 (63.1)	2,054 (74.5)
CTAS, N (%)		
1	4 (0.6)	5 (0.2)
2	241 (34.5)	787 (28.6)
3	406 (58.1)	1,673 (60.7)
4	48 (6.9)	291 (10.6)
5	0 (0)	0 (0)
Arrival by Ambulance, N (%)	321 (45.9)	962 (34.9)
Triage Vital Signs at Index Visit		
Heart Rate, Mean ± SD	77.6 ± 15.6	82.1 ± 21.8
Systolic Blood Pressure, Mean ± SD	149 ± 24.8	140 ± 27.6
Diastolic Blood Pressure, Mean ± SD	84.2 ± 14.0	82.6 ± 23.3
Blood Glucose, Mean ± SD	7.45 ± 2.41	7.39 ± 2.96
Other Imaging Ordered at Index Visit, N (%)		
NCCT	20 (2.9)	519 (18.8)
MRI	26 (3.7)	16 (0.6)
MRA	1 (0.1)	0 (0)
Index Visit ED Disposition		
Discharge Home, N (%)	530 (75.8)	2427 (88.1)
Hospital Admission, N (%)	166 (23.7)	316 (11.5)
Index Visit Stroke Team Consult in ED, N (%)	347 (49.6)	496 (18.0)
Index Visit Referral to Stroke Prevention Clinic, N (%)	132 (18.9)	9 (0.3%)
Any Neuroimaging Within 30-Days (Including Index Visit Imaging), N (%)	699 (100)	707 (25.7)

Index visit CTA and 30-day outcomes of interest

30-day stroke or TIA diagnosis was found in 145 patients (20.7%) of the CTA group, and 32 patients (1.2%) in the No CTA group. The unadjusted OR for this association was 22.3 (95% CI: 15.03-33.02) (Table 2). After adjusting for predefined baseline characteristics, stroke risk factors, and presentation acuity, the adjusted OR was found to be 18.3 (95% CI: 14.85-22.45). Among patients with 30-day diagnosis of stroke or TIA, the proportion of diagnoses made at index ED visit for the CTA vs No CTA groups was 82.8% (N=120) and 65.6% (N=21) respectively.

**Table 2:** Summary of the association between CTA and 30-day diagnosis of stroke or TIA *Adjusted according to: age, sex, previous stroke history, CTAS score, ambulance arrival, site of index ED visit, systolic blood pressure at triage, additional neuroimaging modalities ordered at index visit. OR: Odds ratio; CI: Confidence interval; CTA: CT with angiography; CTAS: Canadian Triage and Acuity Scale; ED: Emergency department; TIA: Transient ischemic attack

Comparative Association Between Index Visit CTA and 30-day Diagnosis of Stroke/TIA	
Unadjusted OR (95% CI)	22.3 (15.03-33.02)
Adjusted OR* (95% CI)	18.3 (14.85-22.45)

The ED LOS for the CTA and No CTA groups were 443 minutes (SD: 169 minutes) and 329 minutes (SD: 164 minutes) respectively (P < 0.001) (Table 3). The CTA and No CTA group had a mean hospital LOS of 6.1 days (SD: 7.3 days) and 8.3 days (SD: 7.8 days), respectively (P < 0.001). In the CTA group, 28 people (4.0%) revisited the ED within 30 days compared to 42 (1.5%) in the No CTA group.

**Table 3:** Secondary ED and hospital outcomes of interest between the No CTA and CTA group CTA: CT with angiography; ED: Emergency department; LOS: Length of stay

	Index Visit CTA Group, N = 699	No Index Visit CTA Group, N = 2756
LOS, Mean ± SD		
ED LOS (Arrival to Disposition) (Mins)	443 ± 169	329 ± 164
Hospital LOS Over First 30 Days (Days)	6.1 ± 7.3	8.3 ± 7.8
1 or More ED Revisit Event(s) Within 30 Days, N (%)	28 (4.0)	42 (1.5)

## Discussion

Participants in our study who had CTA ordered at their index ED visit had 18.3 times greater odds of receiving a diagnosis of TIA or stroke at 30 days, even after adjusting for predefined clinically relevant baseline variables such as age, sex, acuity of presentation, and stroke or TIA risk factors. This likely reflects the clinical gestalt of ED physicians in assessing pre-test probability for stroke or TIA. The findings indicate a mixed impact of CTA utilization on healthcare system flow. For patients in the CTA group, there was almost a two-hour increase in ED LOS on average and 2.5% increase in frequency of 30-day ED revisit events. However, there was also a two-day reduction in hospital LOS among admitted patients and a reduction in delayed diagnosis among those who ultimately received a 30-day diagnosis of stroke or TIA. The increased ED LOS is likely impacted by the time required to complete and interpret imaging in patients who received index visit CTA, and the increased revisit frequency may be explained by incidental findings requiring follow-up, patient anxiety, or persistent or recurrent symptoms. In contrast, the two-day reduction in hospital LOS among admitted patients may be explained by expedited diagnosis and treatment as a result of index CTA imaging.

Clinical implications

Risk stratification of undifferentiated vertigo remains challenging in the ED. Our data appears to support clinician gestalt in neuroimaging utilization for patients at overall higher risk of 30-day stroke or TIA diagnosis. However, approximately four out of five index visit CTAs were not associated with 30-day stroke or TIA diagnosis and represent a potential target for reducing unnecessary imaging and cost to the system.

Comparison to previous studies and research implications

Studies have shown that timely treatment of ischemic stroke is key to better functional outcomes post stroke [10-12]. Therefore, accurate and early diagnosis of stroke is essential. PCIS is especially challenging to diagnose due to often non-specific symptoms, such as vertigo and/or altered mentation [13]. Existing literature identifies limited sensitivity of NCCT imaging in the posterior fossa of the brain as one of the contributors to PCIS misdiagnosis [14,15]. The DOUBT trial found that up to 30% of patients with low-risk TIA or minor stroke symptoms with initially negative CT scans had ischemic lesions identified on subsequent MRI [16]. Given the challenging nature of PCIS diagnosis in the ED, and cost barriers to MRI access, our study evaluates the utility of CTA as an accessible adjunct in vertigo risk stratification through the inclusion of vascular imaging.

The role of CTA in acute ischemic stroke has been previously assessed as it relates to evaluating collateral circulation and predicting the efficacy of endovascular treatment options [17]. Other studies support the predictive value of CTA for short-term stroke and TIA risk stratification through identification of clinically important vascular abnormalities [9]. Our study explores the implications of CTA utilization in undifferentiated ED vertigo patients from a patient-centered perspective in terms of association with 30-day diagnostic outcomes and ED and hospital resource considerations.

This study expands current evidence regarding utilization of CTA for stroke diagnosis in the ED, and considers the impact of neuroimaging choice on healthcare system resource utilization, balancing earlier diagnosis and shortened hospital LOS against prolonged ED LOS and increased ED revisits. Further work is required to more clearly differentiate high risk versus low risk vertigo ED subgroups based on clinical factors to inform when neuroimaging should be utilized versus when it can be safely avoided.

Strengths and limitations

The strengths of this study include the large sample size, robust database linkage, adjustment for relevant clinical baseline characteristics, and consideration of clinically relevant secondary outcomes of interest. However, due to the retrospective nature of this study and reliance on administrative data, certain variables could not be accounted for, including physical exam findings or associated neurologic deficits, degree of symptom severity, type of vertigo (i.e., continuous or episodic), and certain patient characteristics that were unreliably coded in the administrative data (e.g., smoking status).

## Conclusions

Our findings indicate that index visit CTA utilization among undifferentiated vertigo patients in a Canadian ED setting is associated with 18.3 times greater odds of 30-day stroke or TIA diagnosis, even after adjusting for factors such as age, sex, prior stroke history, and clinical acuity. This likely reflects appropriate risk stratification based on ED clinician gestalt. However, up to four out of five ED-ordered CTAs in this population are potentially avoidable. CTA utilization in undifferentiated vertigo remains heterogeneous and has a mixed impact on healthcare system flow, with increased ED LOS and 30-day ED revisit events, but decreased hospital LOS and reduced delays in stroke or TIA diagnosis. Further study is required to determine which specific imaging findings are most predictive of downstream stroke or TIA risk, and when neuroimaging may be safely avoided.

## Appendices

Appendix 1 

**Table 4:** Glossary of variable definitions ED: Emergency department; TIA: Transient ischemic attack; LOS: Length of stay; CTA: CT with angiography

Variable Name	Definition	Calculation Formula
30-Day Diagnosis of Stroke/TIA	Assigned when one of the following 2 criteria were satisfied: 1. Discharge diagnosis on DAD (inpatient database) of TIA, ischemic stroke, or hemorrhagic stroke on: a) the first inpatient encounter at index inpatient episode of care; or b) subsequent inpatient episode of care within 30 days (from earliest ED imputed arrival date) of the index ED visit 2. Diagnosis on NACRS database of minor stroke or TIA on the last ED encounter of: a) the index ED episode of care; or b) a subsequent ED episode of care within 30 days of the index ED visit.	N/A
ED LOS	LOS in hours was calculated for patients who: 1. Visited the ED and were subsequently admitted as an inpatient to the same reporting facility; or 2. Visited the ED and were subsequently discharged or transferred.	(ED Disposition Date/Time) – (Registration Time or Triage Time) (ED Departure Date/Time) – (Registration Time or Triage Time)
Repeat ED Visits	At least one documented return visit to a Calgary ED for any cause within 30 days following index visit.	Percentage of return for each group (CTA and No CTA) was calculated as the proportion of patients who revisited the ED within 30 days
Total Hospital LOS	Total patient days in hospital (ED or inpatient) in the 30-day period from index visit arrival.	ED LOS + Acute LOS (defined as the number of acute care days from the date of admission to the date of discharge)
